# Differential Modulation of Spinal Angiotensin-Converting Enzymes Plays a Critical Role in the Development of Trigeminal Neuropathic Pain

**DOI:** 10.3390/ph19050764

**Published:** 2026-05-13

**Authors:** Jo-Young Son, Yu-Mi Kim, Song-Hee Kang, Jin-Sook Ju, Dong-Kuk Ahn

**Affiliations:** Department of Oral Physiology, Craniofacial Nerve-Bone Network Research Center, School of Dentistry, Kyungpook National University, Daegu 41940, Republic of Korea

**Keywords:** angiotensin, angiotensin converting enzyme, antinociception, trigeminal neuralgia, trigeminal nucleus caudalis

## Abstract

**Background/Objectives:** While the functions of angiotensin-converting enzyme (ACE) 1 and 2 are well established in peripheral tissues, the role of the spinal ACE1 and ACE2 pathways in the development of neuropathic pain remains unclear. This study examined the role of the spinal ACE1 and ACE2 pathways in trigeminal neuropathic pain produced by inferior alveolar nerve (IAN) injury. **Methods:** The experiments were conducted using male Sprague-Dawley rats (6–8 weeks old, weighing 220–250 g). The left mandibular second molar was extracted, and a dental mini-implant was placed to induce IAN injury. IAN injury produced robust and long-lasting mechanical allodynia and markedly increased angiotensinogen (AGT) expression within the ipsilateral trigeminal subnucleus caudalis (iTSC). **Results:** Neuropathic mechanical allodynia was inhibited by intracisternally administered losartan (an angiotensin II type-1 receptor antagonist), but not by an angiotensin II type-2 receptor antagonist. Intracisternal treatment with captopril (an ACE1 inhibitor) and diminazene aceturate (an ACE2 activator) produced significant anti-allodynic effects. Intracisternally injected angiotensin-(1-7) reduced neuropathic mechanical allodynia, and this anti-allodynic effect was blocked by pretreatment with A779, a Mas receptor inhibitor. In naïve rats, the intracisternal administration of DX600 (an ACE2 inhibitor) resulted in mechanical allodynia, which was inhibited by intracisternal pretreatment with losartan. IAN injury led to upregulated ACE1 expression and downregulated ACE2 expression in the iTSC. **Conclusions:** Our findings indicate that IAN injury induces a polarized shift in the ACEs within the iTSC, characterized by increased ACE1 and decreased ACE2 expression. Their modulation may therefore offer a promising strategy for developing effective treatments for chronic pain.

## 1. Introduction

The renin–angiotensin system (RAS) participates in the regulation of vascular resistance, balance of fluid and electrolytes, and hormone secretion [1,2]. Angiotensin (Ang) II, one of the major bioactive components of the RAS, has two receptor types: Ang II type-1 (AT1) and Ang II type-2 (AT2) receptors [3]. Emerging evidence supports the participation of Ang II in the processing of pain information. Ang II is present in the dorsal root ganglia, which contain the cell bodies of primary afferent neurons, in both humans and rats [4,5,6]. Moreover, Ang II shows colocalization with the immunoreactivity of calcitonin gene-related peptide (CGRP) and substance P, immunoreactivity—two key neuropeptides involved in nociceptive signaling—in the dorsal root ganglia of both humans and rats [6]. These experimental results suggest that Ang II modulates pain transmission in primary afferent neurons.

Angiotensin-converting enzyme (ACE) 1 converts Ang I, which is generated from angiotensinogen (AGT), into Ang II [7]. Conversely, ACE2 catalyzes the degradation of Ang I or Ang II to form angiotensin 1-7 [Ang-(1-7)], which activates the Mas receptor and exerts antihypertensive effects, thereby counteracting the actions of ACE1 [8]. These experimental findings indicate that the ACE1 and ACE2 pathways play crucial roles in Ang II–mediated actions within peripheral tissues.

Peripheral Ang II, regulated by the ACE1 and ACE2 pathways, is unable to influence the central nervous system (CNS) because it cannot cross the blood–brain barrier. Therefore, the presence of ACE-related substances within the CNS is essential for Ang II to exert its biological actions. Previous studies have demonstrated an extensive ACE distribution in the brain and the presence of AGT in astrocytes and cerebrospinal fluid [9,10,11]. Moreover, intrathecally injected Ang II results in remarkable nociceptive behaviors, such as biting or licking the hind paw [12]. Recently, the intracisternal injection of Ang II produced allodynia and hyperalgesia in the facial area [13]. Additionally, intrathecal administration of losartan, an AT1 receptor antagonist, showed significant alleviation of nociceptive behavior in the formalin test in mice [14]. These results imply that ACE must exist in the CNS to generate Ang II, which modulates pain processing. However, little research shows that ACE modulates the processing of neuropathic pain in the CNS.

The present study investigated the role of spinal ACEs in trigeminal neuropathic pain induced by inferior alveolar nerve (IAN) injury. Specifically, we examined the alterations in AGT expression within the ipsilateral trigeminal subnucleus caudalis (iTSC) and evaluated the effects of inhibiting the ACE1 pathway following IAN injury. The contribution of the ACE2 pathway to neuropathic pain was also assessed by either activating this pathway or via the intracisternal administration of Ang-(1-7). Furthermore, the effects of blocking the ACE2 pathway on pronociceptive behavior were evaluated in naïve rats. Finally, the study analyzed the expression levels of ACE1 and ACE2 subsequent to IAN injury.

## 2. Results

**Figure 1:**
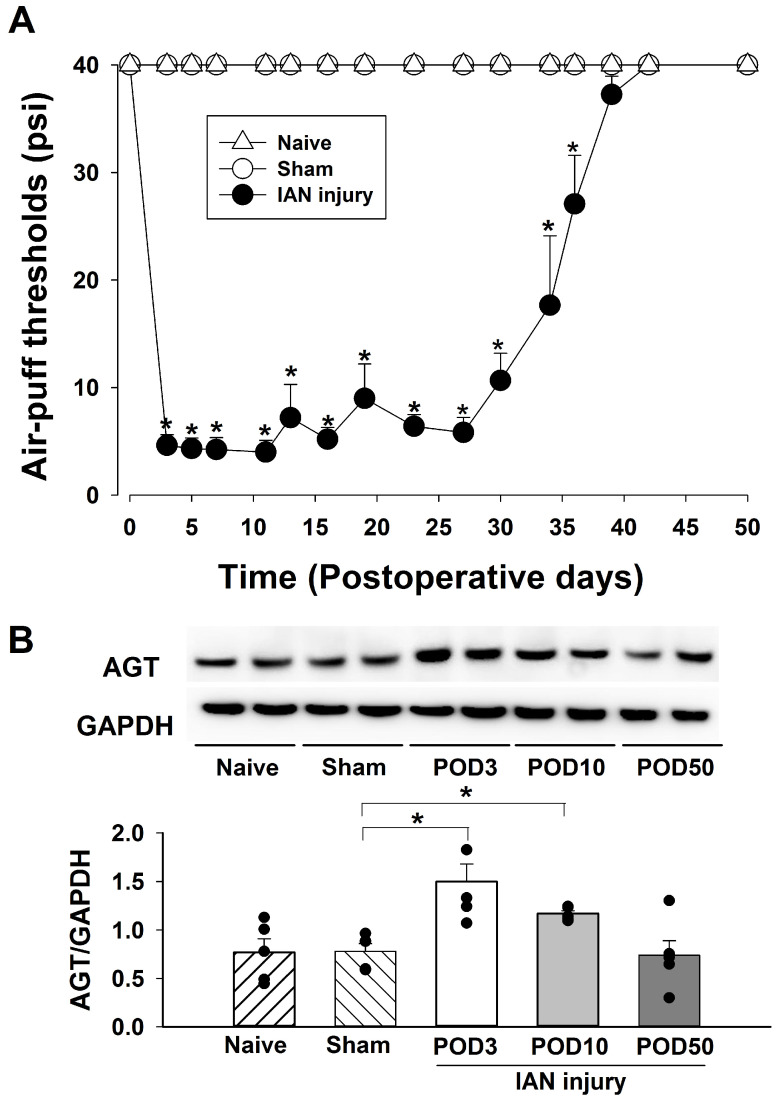
Time-course analysis of alterations in air-puff thresholds and angiotensinogen (AGT) expression following inferior alveolar nerve (IAN) injury caused by a mal-positioned dental implant. (**A**) IAN injury caused significant trigeminal mechanical allodynia compared to the sham-operated group. Mechanical allodynia was observed on postoperative day (POD) 3 and persisted until POD 36. The cut-off pressure was 40 psi. Data are displayed as mean ± SEM, followed by repeated measures ANOVA with Holm–Sidak post hoc tests. * Symbol shows the significance between the sham and IAN injury groups (*p* < 0.05). There were 7 animals in each group. (**B**) IAN injury significantly upregulated AGT expression in the ipsilateral trigeminal subnucleus caudalis (iTSC) on PODs 3 and 10, compared to the sham-treated group. AGT expression levels returned to normal values on POD 50. Data are displayed as mean ± SEM, followed by one-way ANOVA, followed by Holm–Sidak post hoc tests. * Symbol shows the significance between the sham and IAN injury groups (*p* < 0.05). There were five animals in each group.

Figure 1 depicts alterations in the air-puff withdrawal thresholds and angiotensin expression following IAN injury caused by misplaced dental implants. We observed that IAN injury significantly caused long-term nociceptive behavior in rats. Rats with IAN injuries exhibited significantly lower air-puff withdrawal thresholds compared to that in sham-operated controls (F_2,15_ = 843.97, *p* < 0.05, *n* = 7/group, Figure 1A). Mechanical allodynia was evident on POD 3 and persisted through POD 36. This study also examined AGT expression in the iTSC on PODs 3, 10, and 50. IAN injury significantly upregulated AGT expression on PODs 3 and 10 compared to the sham-treated group (*p* < 0.05, *n* = 5/group, Figure 1B). The AGT expression levels returned to normal values on POD 50.

**Figure 2:**
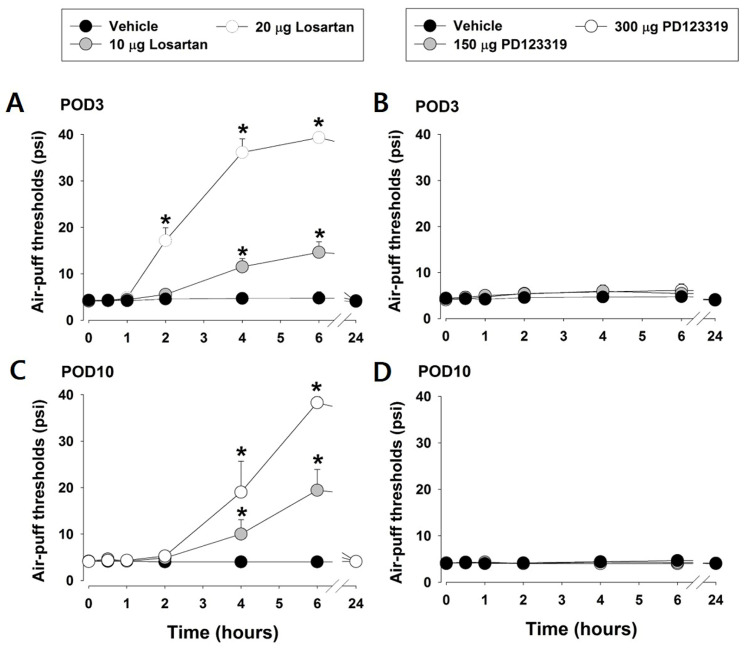
Effects of losartan, an angiotensin type 1 (AT1)-receptor antagonist, and PD123319, an AT2-receptor antagonist, on mechanical allodynia induced by inferior alveolar nerve (IAN) injury. (**A**,**C**) On postoperative days (PODs) 3 and 10, intracisternal administration of losartan (10 or 20 μg) significantly alleviated neuropathic mechanical allodynia compared to the vehicle-treated group. Data are displayed as mean ± SEM, followed by repeated measures ANOVA with Holm–Sidak post hoc tests. * Symbol shows the significance between the vehicle and losartan-treated groups (*p* < 0.05). There were 7 animals in each group. (**B**,**D**) However, intracisternal administration of PD123319 did not affect mechanical allodynia. Data are displayed as mean ± SEM. There were 7 animals in each group.

This study examined the participation of AT1 and AT2 receptors in the development of neuropathic pain. Figure 2 depicts the effects of losartan and PD123319 on mechanical allodynia caused by a mispositioned dental implant. On POD 3, intracisternally administered losartan (10 or 20 μg) significantly alleviated mechanical allodynia compared with the vehicle treatment (F_2,21_ = 108.117, *p* < 0.05, *n* = 7/group, Figure 2A). Moreover, intracisternally injected losartan also attenuated mechanical allodynia 10 days post-IAN injury (*p* < 0.05, *n* = 7/group, Figure 2C). However, intracisternally injected PD123319 had no significant effect on mechanical allodynia (*n* = 7/group, Figure 2B,D).

**Figure 3:**
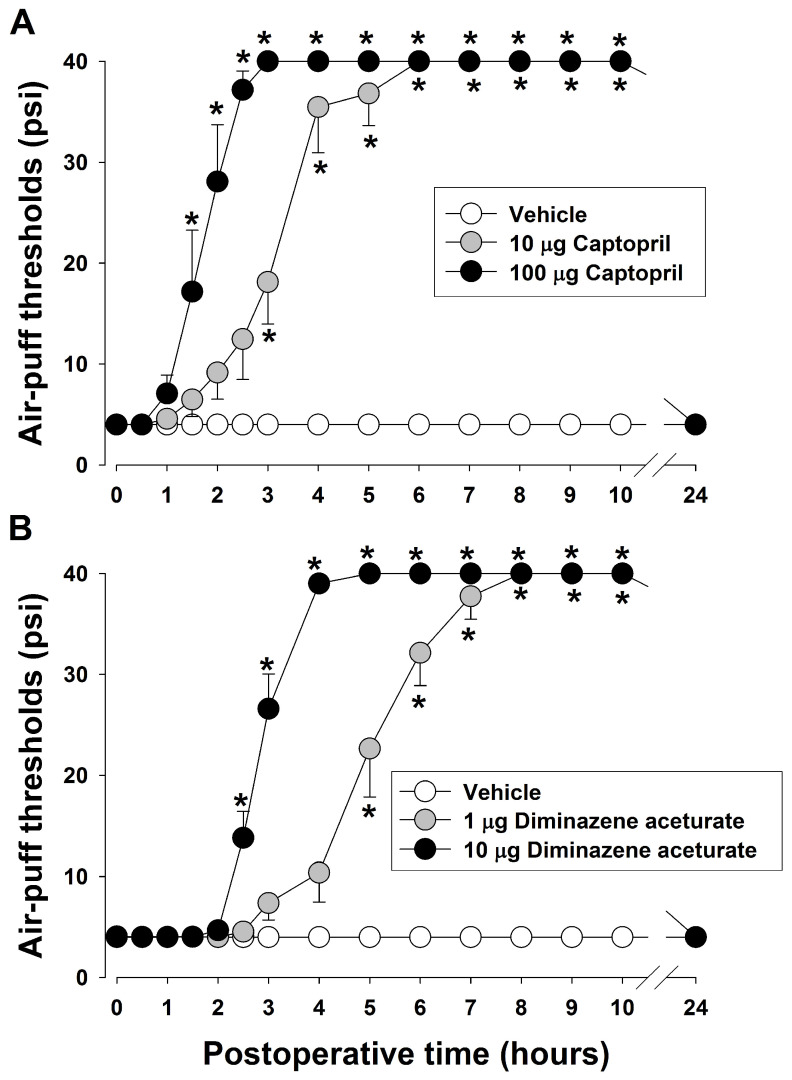
Effects of blocking the spinal angiotensin-converting enzyme (ACE)1 pathway and accelerating the ACE2 pathway on mechanical allodynia induced by inferior alveolar nerve (IAN) injury. (**A**) To block the ACE1 pathway, captopril, an ACE1 inhibitor, was administered intracisternally on postoperative day (POD) 7. Intracisternal treatment with captopril, 10 or 100 µg, produced significant anti-allodynic effects compared to vehicle treatment. Data are displayed as mean ± SEM, followed by repeated measures ANOVA with Holm–Sidak post hoc tests. * Symbol shows the significance between the vehicle and captopril-treated groups (*p* < 0.05). There were 7 animals in each group. (**B**) Intracisternal injection of diminazene aceturate (1 or 10 µg), an ACE2 activator, produced significant anti-allodynic actions compared to vehicle treatment. Data are displayed as mean ± SEM, followed by repeated measures ANOVA with Holm–Sidak post hoc tests. * Symbol shows the significance between the vehicle and diminazene aceturate-treated groups (*p* < 0.05). There were 7 animals in each group.

Figure 3 illustrates the alterations in mechanical allodynia after the modulation of spinal ACE1 and ACE2 pathways. To block the ACE1 pathway, captopril (an ACE1 inhibitor) was administered intracisternally on POD 7. Intracisternal treatment with captopril (10 or 100 µg) significantly produced anti-allodynic effects compared to vehicle treatment (F_2,15_ = 178.236, *p* < 0.05, *n* = 7/group, Figure 3A). The anti-allodynic effects occurred 1.5 h following the injection of 100 µg of captopril and lasted for up to 10 h. This study also tested the action of diminazene aceturate (an ACE2 activator) on mechanical allodynia on POD 7. Intracisternal administration of diminazene aceturate (1 or 10 μg) produced significant anti-allodynic effects compared with vehicle treatment (F_2,15_ = 146.890, *p* < 0.05, *n* = 7/group, Figure 3B). The anti-allodynic effect of 10 μg diminazene aceturate emerged 2.5 h after administration and persisted for up to 10 h.

**Figure 4:**
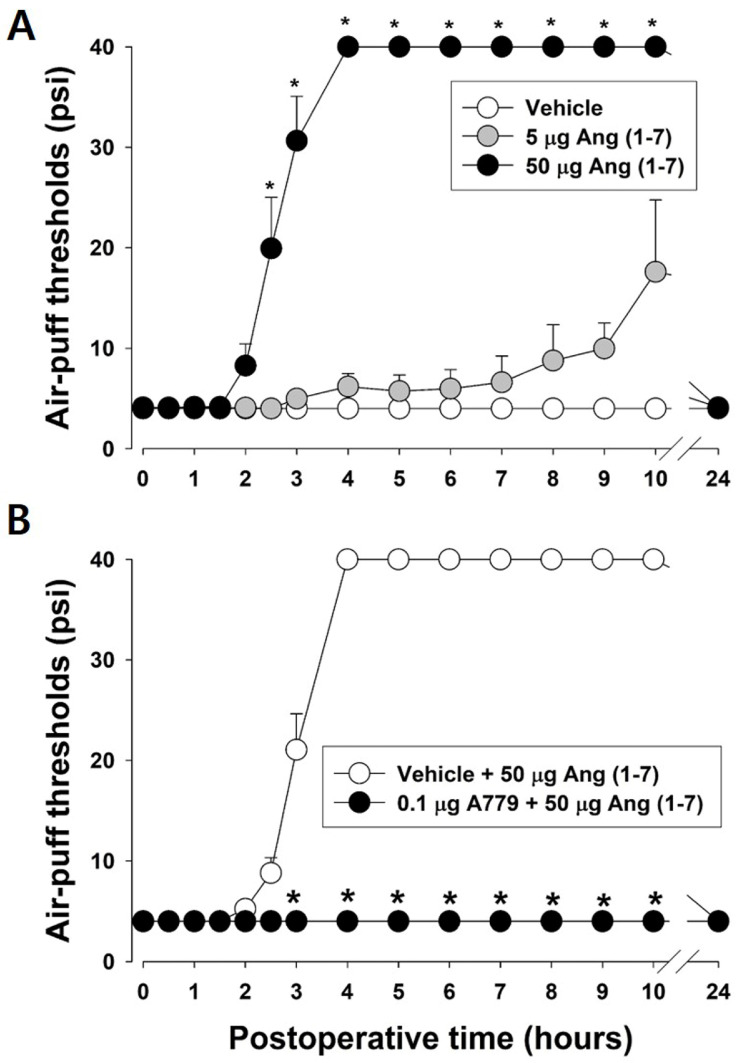
Effects of intracisternally injected angiotensin (Ang)-(1-7) on mechanical allodynia induced by inferior alveolar nerve (IAN) injury. (**A**) On postoperative day (POD) 7, intracisternal injection of 50 µg of Ang-(1-7) significantly attenuated neuropathic mechanical allodynia, whereas a low dose (5 µg) of Ang-(1-7) did not affect mechanical allodynia. Data are displayed as mean ± SEM, followed by repeated measures ANOVA with Holm–Sidak post hoc tests. * Symbol shows the significance between the vehicle and Ang (1-7)-treated groups (*p* < 0.05). There were 7 animals in each group. (**B**) To confirm the antinociceptive effects of Ang-(1-7) through Mas receptor, A779 (a Mas-receptor inhibitor) was administered 3 h before the Ang-(1-7) injection. Pretreatment with A779 significantly blocked the antinociceptive effects of Ang-(1-7) compared to vehicle treatment. Data are displayed as mean ± SEM, followed by repeated measures ANOVA with Holm–Sidak post hoc tests. * Symbol shows the significance between the vehicle and A779-treated groups (*p* < 0.05). There were 7 animals in each group.

The effects of intracisternally injected Ang-(1-7) on mechanical allodynia induced by IAN injury are depicted in Figure 4 to investigate an alternative pathway of Ang. On POD 7, intracisternally injected 50 µg Ang-(1-7) significantly alleviated neuropathic mechanical allodynia (F_2,15_ = 146.89, *p* < 0.05, *n* = 7/group, Figure 4A), whereas a low dose (5 µg) of Ang-(1-7) did not affect neuropathic mechanical allodynia. To confirm the antinociceptive effects of Ang-(1-7) through the Mas receptor, A779 (a selective Mas-receptor inhibitor) was administered 3 h before the Ang-(1-7) injection. Pretreatment with A779 significantly blocked the antinociceptive effects of Ang-(1-7) compared to vehicle treatment (F_1,10_ = 4111.776, *p* < 0.05, *n* = 7/group, Figure 4B).

**Figure 5:**
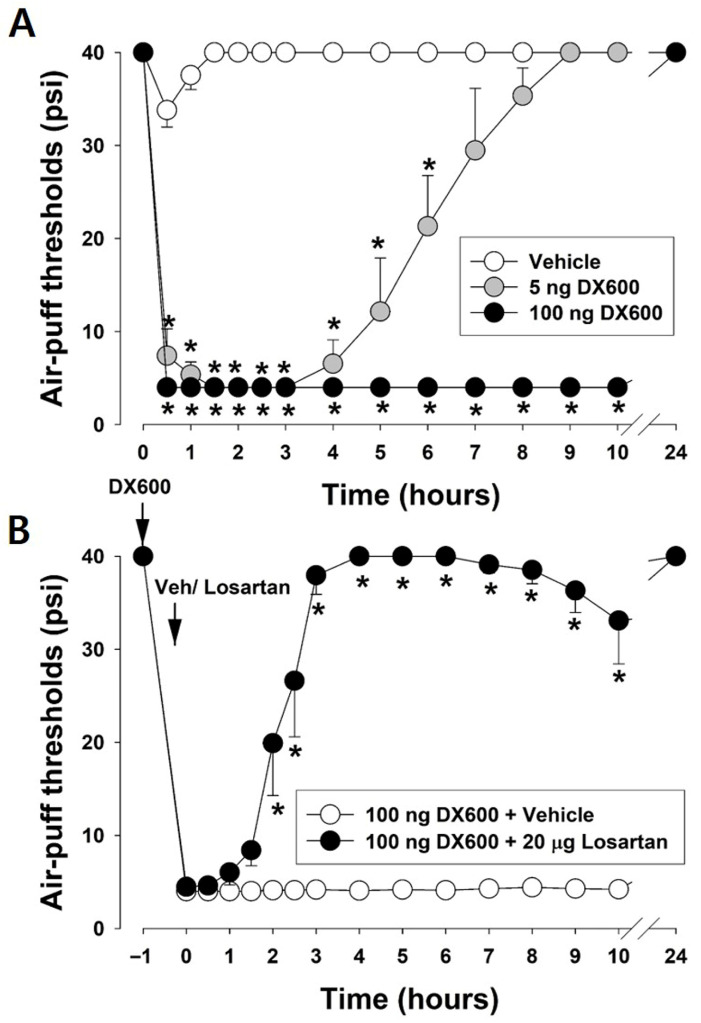
Effects of blocking the angiotensin-converting enzyme (ACE)2 pathway on air-puff thresholds in naïve rats. (**A**) Intracisternal administration of DX600 (5 or 100 ng), an ACE2 inhibitor, induced significant mechanical allodynia in naïve rats. Mechanical allodynia was observed at 0.5 h and maintained for 10 h after intracisternal injection of 100 ng of DX600. Data are displayed as mean ± SEM, followed by repeated measures ANOVA with Holm–Sidak post hoc tests. * Symbol shows the significance between the vehicle and DX600-treated groups (*p* < 0.05). There were 7 animals in each group. (**B**) To investigate the role of the angiotensin type 1 (AT1) receptor in the pronociceptive effects caused by blocking the ACE2 pathway, losartan, an AT1 receptor antagonist, was injected intracisternally 3 h after the administration of an ACE2 inhibitor. Intracisternal administration of 20 μg of losartan blocked mechanical allodynia induced by blocking the ACE2 pathway in naïve rats. Data are displayed as mean ± SEM, followed by repeated measures ANOVA with Holm–Sidak post hoc tests. * Symbol shows the significance between the vehicle and losartan-treated groups (*p* < 0.05). There were 7 animals in each group.

Figure 5 depicts the changes in the air-puff thresholds after blocking the ACE2 pathway in naïve rats. The intracisternal administration of DX600 (5 or 100 ng), an ACE2 inhibitor, induced significant mechanical allodynia in naïve rats (F_2,15_ = 314.714, *p* < 0.05, *n* = 7/group, Figure 5A). Mechanical allodynia was observed at 0.5 h and maintained for 10 h after intracisternal injection of 100 ng of DX600. To investigate the role of the AT1 receptor, losartan (an AT1-receptor antagonist) was injected intracisternally 1 h after administration of the ACE2 inhibitor. Intracisternal administration of 20 µg of losartan blocked the mechanical allodynia induced by blocking the ACE2 pathway in naïve rats (F_1,10_ = 216.882, *p* < 0.05, *n* = 7/group, Figure 5B).

**Figure 6:**
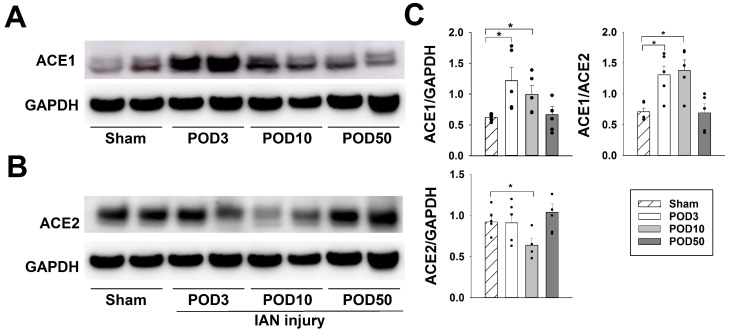
Changes in angiotensin-converting enzyme (ACE)1 and ACE2 expression after inferior alveolar nerve (IAN) injury. (**A**) IAN injury resulted in upregulated ACE1 expression in the ipsilateral trigeminal subnucleus caudalis (iTSC) on postoperative day (POD) 3 and 10 compared to the sham group (*p* < 0.05). On POD 50, ACE1 expression levels returned to the pretreatment level. (**B**) In contrast, IAN injury decreased ACE2 expression on POD 10 compared to the sham group (*p* < 0.05). ACE2 expression returned to the pretreatment level on POD 50. (**C**) ACE1 to ACE2 expression ratio significantly increased following nerve injury and returned to the pretreatment level on POD 50 (*p* < 0.05). Data are displayed as mean ± SEM, followed by one-way ANOVA, followed by Holm–Sidak post hoc tests. * Symbol shows the significance between the sham and IAN injury groups (*p* < 0.05).

Figure 6 illustrates the changes in ACE1 and ACE2 expression post-IAN injury. IAN injury resulted in the upregulation of ACE1 expression in the iTSC on PODs 3 and 10, compared to the sham-operated group (*p* < 0.05, *n* = 5/group, Figure 6A). On POD 50, the ACE1 expression level returned to the pretreatment level. In contrast, IAN injury decreased the ACE2 expression on POD 10 compared to the sham-treated group (*p* < 0.05, *n* = 5/group, Figure 6B). Also, ACE2 expression returned to pretreatment level on POD 50. This study analyzed the comparison of the ACE1 to ACE2 expression ratio following IAN injury. The ACE1/2 expression ratio increased on PODs 7 and 10 following IAN injury (*p* < 0.05, Figure 6C) and returned to the pretreatment level on POD 50.

## 3. Discussion

This study demonstrated the involvement of the spinal ACE pathway in neuropathic pain following nerve injury. Blocking the spinal ACE1 pathway or activating the spinal ACE2 pathway reduced neuropathic mechanical allodynia. Intracisternal injection of Ang-(1-7) attenuated neuropathic mechanical allodynia and pretreatment with a selective Mas receptor antagonist inhibited the antiallodynic effects induced by Ang-(1-7). Furthermore, blockade of the ACE2 pathway produced pronociceptive effects in naïve rats, and pretreatment with losartan (an AT1-receptor antagonist) attenuated these pronociceptive effects. Finally, IAN injury increased ACE1 expression and attenuated ACE2 expression in the iTSC. These results suggest that the spinal ACE pathway contributes to the development of neuropathic pain and that differential expression of ACE1 and ACE2 plays a critical role in neuropathic pain following IAN injury.

### 3.1. Participation of Spinal Ang II via AT1 Receptor in Neuropathic Mechanical Allodynia

Recently, a previous study introduced an AT2-receptor antagonist that was developed as a painkiller [15,16]. EMA401, a highly selective AT2-receptor antagonist, has demonstrated significant analgesic efficacy in patients with postherpetic neuralgia and diabetic neuropathy in randomized, double-blind clinical trials [15,16]. However, systemically administered EMA401 does not cross the blood–brain barrier, which is composed of pericytes, astrocytes, and specialized endothelial cells [17]. Therefore, Ang II should be synthesized within the CNS to activate its receptors. Nonetheless, several previous studies have demonstrated that Ang II contributes to pain transmission in the CNS. Recent studies have shown that intrathecal administration of Ang II elicits nociceptive behavioral responses in mice [12,14] and significant allodynia and hyperalgesia in rats [13]. Although these findings suggest that centrally derived Ang II contributes to pain processing within the CNS, few studies have reported the specific role of spinal Ang II in modulating neuropathic pain following nerve injury. Our study found that IAN injury increased AGT expression in the iTSC, accompanied by mechanical allodynia.

The present study also showed that intracisternally administered losartan, but not PD123319, blocked trigeminal mechanical allodynia induced by IAN injury. These results imply that the AT1 receptor participates in trigeminal neuropathic pain. Previous studies have shown the involvement of the AT1 receptor in pain processing in the CNS. Intrathecally injected losartan blocked mechanical allodynia in rats with streptozotocin-induced diabetic neuropathic pain [18] and attenuated nociceptive scratching behavior in the mouse formalin test by blocking the phosphorylation of p38 MAPK [19]. Moreover, intrathecal injection of Ang II-induced nociceptive behavior was mediated by AT1 receptors on the neurons and astrocytes in the spinal cord of mice [19]. A recent study also reported that intracisternal administration of Ang II produced mechanical allodynia through astroglial AT1 receptors [13]. These results indicate that the spinal AT1 receptor, but not the AT2 receptor, plays a critical role in pain transmission at the level of the spinal cord. However, the AT2 receptor also plays a role in modulating nociceptive processing. The intravenous administration of AT2 receptor antagonists reduced mechanical allodynia in rodent neuropathic pain models [20]. Intraperitoneal administration of a selective AT2 antagonist attenuated peripheral neuropathic pain following a chronic constriction injury of the sciatic nerve [21]. These results suggest that the AT2 receptor also influences nociceptive processing in neuropathic pain. Conversely, intrathecal administration of PD123319 had no effect on streptozotocin-induced tactile allodynia [18]. Therefore, these results suggest that the role of the AT2 receptor in neuropathic pain remains controversial. Another recent study observed that the AT2 receptor is located in CGRP-immunoreactive primary afferent terminals, which regulate thermal hyperalgesia induced by intracisternally injected Ang II [13]. These results, including those of the present study, demonstrate that spinal Ang II plays a crucial role in neuropathic pain; however, its effects are differentially mediated by AT1 and AT2 receptors.

### 3.2. Role of Spinal ACE1 and ACE2 Pathways in Neuropathic Mechanical Allodynia

The primary function of ACE1 is to convert Ang I to Ang II [7], and its presence within the CNS is required to synthesize Ang II at the level of the spinal cord. This study examined changes in neuropathic mechanical allodynia following the modulation of spinal ACE pathways. The intracisternal administration of captopril, an ACE1 inhibitor, significantly produced anti-allodynic effects following IAN injury, suggesting that the centrally activated ACE1 pathway contributes to the development of neuropathic mechanical allodynia after IAN injury. ACE2 is known to convert Ang I or Ang II into Ang-(1-7), which counteracts the effects of ACE1 [8]. This study investigated the changes in neuropathic mechanical allodynia following the activation of the spinal ACE2 pathway. Intracisternal administration of diminazene aceturate, an ACE2 activator, blocked neuropathic mechanical allodynia. Furthermore, intracisternal injection of Ang-(1-7) inhibited neuropathic mechanical allodynia, which was reversed by pretreatment with A779, a Mas receptor inhibitor. Several lines of evidence support the involvement of spinal ACEs in pain processing. Treatment with enalapril, an ACE1 inhibitor, attenuated diabetic neuropathy in streptozotocin-treated rats [22]. The intrathecal injection of ACE1 inhibitors (enalapril or captopril) significantly increased the frequency of paw withdrawal responses to mechanical stimuli [23]. Moreover, the activation of spinal ACE2 inhibited formalin-induced nociceptive behavioral responses in mice [24]. The intrathecal administration of Ang-(1-7) attenuated the nociceptive response via spinal Mas receptor [25,26]. When combined with the present findings, these results suggest that the spinal ACE1 pathway produces pronociceptive effects, while the spinal ACE2 pathway produces antinociceptive effects. Therefore, the spinal ACE2/Ang-(1-7) axis represents a novel antinociceptive mechanism that counteracts the pronociceptive effects of the spinal ACE1/Ang II pathway [8].

### 3.3. Role of Differential Expression of Spinal ACE1 and ACE2 in Neuropathic Mechanical Allodynia

To further investigate the homeostatic role of ACE2, we examined whether its inhibition could induce pain in the absence of nerve injury. Rather than administering the ACE2 inhibitor DX600 to rats with pre-existing IAN injury, we used naive rats to test the hypothesis that blocking ACE2 would be sufficient to trigger nociception. The present study demonstrated that the intracisternal injection of DX600, an ACE2 inhibitor, produced significant mechanical allodynia in naïve rats. Notably, this induced pain was effectively suppressed by losartan, an AT1 receptor antagonist. These findings suggest that diminished ACE2 activity leads to a relative dominance of ACE1-mediated signaling, proving that maintaining a precise balance between these two enzymes is a critical determinant of pain signaling homeostasis. Moreover, these results suggest that both the spinal ACE1 and ACE2 pathways are crucial to neuropathic pain following IAN injury. Furthermore, previous immunofluorescence studies have demonstrated the co-localization of AT1 receptors with the astrocytic marker GFAP in the trigeminal subnucleus caudalis, as well as AT2 receptor expression in CGRP-positive neurons in the trigeminal ganglion [13]. Mechanistically, both ACE1 and ACE2 have been identified in neuronal and glial populations within the central nervous system and are known to play important roles in the regulation of neuroinflammation [27,28,29]. These findings suggest that ACE-related mechanisms involved in pain modulation are likely mediated through complex neuron–glia interactions. However, there is no study on the interrelationship between the ACE1/2 pathways and neuropathic pain. This study reported that IAN injury upregulated ACE1 expression, which returned to the pretreatment level on POD 50. However, nerve injury downregulated the ACE2 expression in the iTSC. The ACE1 to ACE2 expression ratio significantly increased following nerve injury and returned to the pretreatment level on POD 50. These results suggest that treatments for neuropathic pain should target both the spinal ACE1 and ACE2 pathways. Moreover, the spinal ACE2 pathway holds promise for the development of beneficial drugs for treating chronic neuropathic pain in the future.

### 3.4. Clinical Perspectives

Recent clinical investigations have explored the AT2 receptor as a viable target for pain management. For instance, EMA401, a highly selective AT2 receptor antagonist, demonstrated significant analgesic efficacy in patients with diabetic neuropathy and postherpetic neuralgia [15,16]. In this context, accumulating evidence indicates that several clinically used angiotensin receptor blockers (ARBs), including losartan, telmisartan, and candesartan, are capable of penetrating the central nervous system and exerting pharmacological effects within the brain. Previous studies have demonstrated that systemically administered ARBs can cross the blood–brain barrier (BBB) and produce anti-inflammatory and neuroprotective effects by modulating central angiotensin signaling [30]. These findings suggest that ARBs can influence central nervous system function independently of their peripheral blood pressure–lowering effects [30,31] and may therefore contribute to the modulation of pain signaling. Accordingly, therapeutic strategies utilizing systemically administered ARBs with BBB permeability may represent a promising approach for the management of chronic pain.

Nevertheless, a clinically approved analgesic that specifically modulates the Ang II pathway has yet to be realized. Our study addresses this gap by demonstrating that bidirectional modulation of the ACEs axis, characterized by ACE1 inhibition and ACE2 activation, effectively attenuates nerve injury-induced allodynia. Our findings indicate that IAN injury induces a polarized shift in the ACEs within the iTSC, characterized by increased ACE1 and decreased ACE2 expression. Because ACE1 and ACE2 exert antagonistic effects on neuropathic pain, maintaining a precise balance between these enzymes appears vital for orofacial sensory homeostasis.

In addition, both ACE1 and ACE2 are expressed in neuronal and glial cell populations within the central nervous system and are known to play important roles in the regulation of neuroinflammation, suggesting that ACE-related mechanisms in pain modulation involve complex neuron–glia interactions [27,28,29]. This hypothesis is supported by evidence that pharmacologically restoring this balance—either through ACE1 inhibition or ACE2 activation—effectively mitigates mechanical hypersensitivity. This dual-target mechanism offers a promising framework for expanding therapeutic options and potentially mitigating tolerance issues associated with long-term chronic pain management. However, the extent to which temporal fluctuations in ACE expression contribute to established chronic pain and influence therapeutic outcomes remains an area for future investigation.

### 3.5. Limitations

While acknowledging the importance of sexual dimorphism in pain research, male SD rats were selected for this study to standardize behavioral responses and eliminate the influence of hormonal cycles on pain outcomes. This choice is supported by literature suggesting that sex hormones significantly alter pain thresholds and treatment efficacy [32,33,34]. Although excluding females may limit the generalizability of these findings, it was a necessary step to minimize experimental variance during this mechanistic evaluation. Further research incorporating both sexes is required to elucidate sex-specific dynamics of observed pain modulation. The sample sizes used in this study (*n* = 7 for behavioral tests and *n* = 5 for Western blot analysis) were determined in accordance with the principles of the 3Rs (Replacement, Reduction, and Refinement), balancing ethical considerations with experimental reliability. Although this study primarily relied on protein-level analyses, the results were consistent with the behavioral findings, thereby strengthening the overall interpretation of the data.

## 4. Materials and Methods

### 4.1. Animals

The study used 242 male Sprague Dawley rats (6–8 weeks old, weighing 220–250 g). The animals were maintained under stable environmental conditions, with a constant temperature and a 12 h light–dark cycle. Water and food were provided ad libitum. For behavioral assessments, 7 animals per group were used, while 5 animals per group were allocated for Western blot analysis. All subjects were randomly assigned to their experimental cohorts. All procedures using animals were approved by the Animal Experimentation Ethics Committee of Kyungpook National University (Approval code: KNU 2023-0048). This study was conducted in accordance with the ethical standards and guidelines established by the International Association for the Study of Pain and the National Institutes of Health (NIH, Bethesda, MD, USA) for the investigation of experimental pain in conscious experimental animals. All procedures were performed under blinded conditions to minimize observer bias.

### 4.2. Neuropathic Pain Animal Model

**Figure 7:**
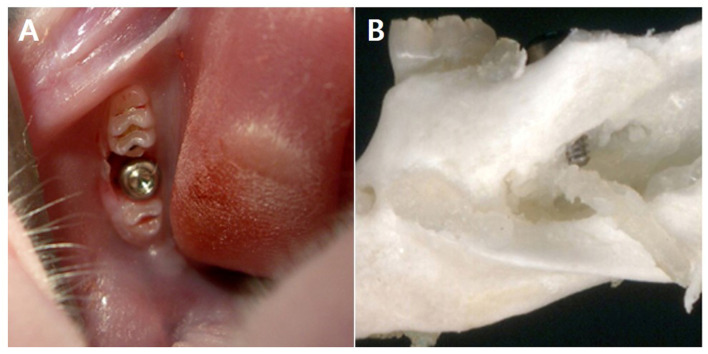
Application of a mini dental implant in a rat. (**A**) Illustration of the crown and root structure of a molar tooth. A dental implant was placed into the alveolar socket following the extraction of the mandibular second molar. (**B**) Penetration of the inferior alveolar canal resulting from improper placement of the dental implant.

Experimental rats were anesthetized with an intramuscular injection of ketamine (40 mg/kg) and xylazine (4 mg/kg) solution. The left lower second molar tooth was extracted under anesthesia. The IAN was intentionally damaged by placing a mini dental implant measuring 1 mm in diameter and 4 mm in length (donated by MegaGen, Daegu, Republic of Korea; Figure 7) [35,36]. In the control group, the second molar tooth was extracted; however, no implants were placed. Following completion of the behavioral assessments, animals were euthanized under deep anesthesia. The final post-experimental analysis included data obtained from rats in which the mini dental implants had accurately targeted and damaged the IAN.

### 4.3. Intracisternal Catheterization

**Figure 8:**
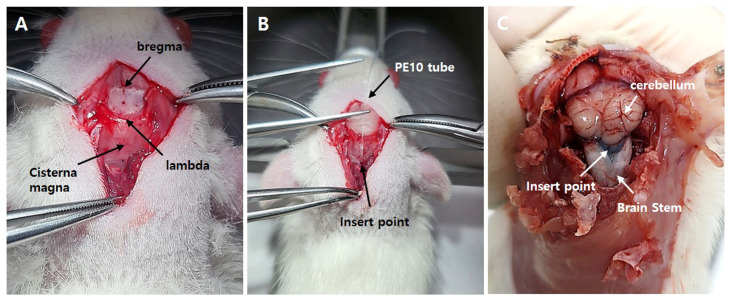
Schematic diagram of intracisternal catheterization. (**A**) The anesthetized rat was secured in a stereotaxic frame, and the skull was exposed. (**B**) A PE-10 cannula was inserted through a small opening in the atlanto-occipital membrane and dura mater using a 25-gauge needle. (**C**) The distribution of the injected solution was confirmed via pontamine sky blue dye injection.

The experimental rats were anesthetized using the same anesthetic drug and fixed in a stereotaxic frame. A small hole was made in the atlanto-occipital membrane at the level of the obex using a 29-gauge needle, as previously described [13,37,38]. A polyethylene tube (PE10; Clay Adams, Parsippany, NJ, USA) was inserted into the small hole. Following implantation, the distal end of the PE10 tube was tunneled subcutaneously and exteriorized at the cranial region (Figure 8). Subsequently, it was securely fixed to a stainless-steel screw anchored in the skull using dental acrylic resin to ensure stable positioning throughout the experimental period. The final analysis excluded animals exhibiting abnormal motor function after catheter insertion or incorrect catheter placement. As shown in previous studies [13,39,40], experimental animals were given a 72 h postoperative recovery period before the initiation of experimental procedures.

### 4.4. Assessment of Orofacial Mechanical Allodynia

To assess mechanical allodynia, each rat was individually housed in a custom-fabricated transparent acrylic chamber located in a dark, sound-attenuated room. In this study, an air-puff method was used to quantitatively assess mechanical allodynia in the facial region. This approach provides reproducible, non-contact stimulation, thereby minimizing stress and potential tissue damage associated with conventional contact-based methods. Previous studies have validated this technique as a reliable measure of neuropathic pain following IAN injury [35], and it has been widely used in experimental models of orofacial pain [41,42,43]. The experimental rats were allowed to acclimate to the environment for at least 30 min before the start of the experiment. Behavioral withdrawal responses were observed following ten consecutive applications of a constant air-puff stimulus (10-s interstimulus intervals, 4-s duration), as previously described [43,44,45]. The air-puff stimulus was delivered at a distance of 1 cm from the skin surface at a 90° angle using a 26-gauge metal tube. Pain sensitivity was quantified as the pressure intensity that elicited withdrawal responses in 50% of air-puff trials. The duration and intensity of the air-puff stimuli were precisely controlled and calibrated using a Pico-Injector system (Harvard Apparatus, Holliston, MA, USA). The air-puff stimuli were delivered at pressures not exceeding 40 psi to avoid potential tissue damage or other adverse effects [13,39,40,46]. Naïve rats exhibited no withdrawal responses to air-puff pressures below this threshold.

### 4.5. Western Blotting

Under euthanasia with the same anesthetic solution (ketamine [1000 mg/kg] and xylazine [100 mg/kg]), tissue sections were obtained from the iTSC on postoperative days (PODs) 3, 10, and 50 following the previously described protocol [47]. The iTSC tissues were homogenized and lysed in T-PER buffer (Thermo Fisher Scientific, Waltham, MA, USA) containing phosphatase and protease inhibitors and centrifuged at 13,000 rpm for 30 min at 4 °C. The protein concentration was quantified using Qubit (Thermo Fisher Scientific), and each investigated marker was normalized to the total protein stain. Equal amounts of protein (30 μg) were loaded onto 3–8% gradient NuPAGE Tris-Acetate gels (Invitrogen, Waltham, MA, USA) and subsequently transferred onto nitrocellulose membranes. The transferred membranes were blocked at room temperature for 1 h in 5% skim milk prepared in tris-buffered saline containing 0.1% Tween 20. The blocked membranes were incubated overnight at 4 °C with the following primary antibodies: AGT (1:1000; cat. no. ab213705; 53 kDa; Abcam, Cambridge, MA, USA), ACE1 (1:10,000; cat. no. ab254222; 180 kDa; Abcam), ACE2 (1:1000; cat. no. ab15348; 105 kDa; Abcam), and glyceraldehyde-3-phosphate dehydrogenase (GAPDH; 1:10,000; cat. no. sc-32233; 37 kDa; Santa Cruz Biotechnology, Dallas, TX, USA). After incubation, the membranes were incubated with horseradish peroxidase (HRP)-conjugated secondary antibodies (1:5000; Bio-Rad, Hercules, CA, USA) for 1 h at room temperature. After the membranes were washed, the protein bands were detected using enhanced chemiluminescence (ECL) substrates (Millipore, Burlington, MA, USA) with a chemiluminescence detection system (Amersham Imager 600, GE Healthcare, Piscataway, NJ, USA). The intensity of specific bands was quantified using ImageJ analysis software, version 1.53t (NIH).

### 4.6. Drugs

Losartan, an AT1-receptor antagonist; PD123319, an AT2-receptor antagonist; and A779 (a Mas-receptor inhibitor) were purchased from Tocris (Bristol, UK). Captopril (an ACE1 inhibitor) and diminazene aceturate (an ACE2 activator) were purchased from Sigma Aldrich (St. Louis, MO, USA). Ang-(1-7) was procured from the Peptide Institute (Tokyo, Japan), and DX600 (an ACE 2 inhibitor) was obtained from Selleckchem (Houston, TX, USA). All chemicals were dissolved in sterile saline, except diminazene aceturate, which was dissolved in 5% dimethyl sulfoxide (DMSO). The pharmacological agents and dosages selected for this study were based on established literature [13,23,24,48,49]. The 5% DMSO vehicle exhibited no detectable influence on pain-related behaviors.

### 4.7. Experimental Protocols

#### 4.7.1. Experimental Design and Timeline

**Figure 9:**
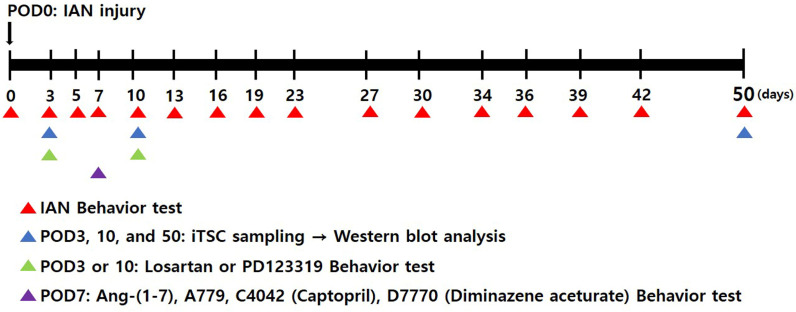
Experimental design and timeline. The diagram specifies the exact time points for surgical interventions, pharmacological administration (intracisternal injection), and tissue collection for Western blot analysis.

The schematic representation illustrates the experimental protocol, outlining the chronological sequence of the study (Figure 9). The diagram specifies the exact time points for surgical interventions, pharmacological administration (intracisternal injection), and tissue collection for Western blot analysis.

#### 4.7.2. The Participation of Spinal Ang II in Trigeminal Neuropathic Pain

This study examined alterations in the air-puff withdrawal thresholds following IAN injury. The air-puff thresholds were monitored at 0, 3, 5, 7, 11, 13, 16, 19, 23, 27, 30, 34, 36, 39, 42, and 50 days after nerve injury (*n* = 7 animals per group). AGT expression in the iTSC was examined on PODs 3, 10, and 50 (*n* = 5 animals per group). In this study, POD 3 was selected to represent the early phase of neuropathic pain, whereas POD 10 was chosen to represent the established phase. This selection was based on previous studies demonstrating the development of significant pain hypersensitivity following nerve injury, accompanied by dynamic changes in microglial and astrocytic activation within the central nervous system [18,50]. To identify the Ang receptors that regulate trigeminal neuropathic pain, losartan (10 or 20 µg/10 µL), an AT1 receptor antagonist or PD123319 (150 or 300 µg/10 µL), an AT2 receptor antagonist, was intracisternally administered on PODs 3 and 10. Alterations in the air-puff thresholds were measured at 0.5, 1, 2, 4, 6, and 24 h following the injection of either vehicle or AT1/AT2 receptor antagonists (*n* = 7 animals per group).

#### 4.7.3. Role of Spinal ACE1 and ACE2 in Trigeminal Neuropathic Pain

ACE1 and ACE2 catalyze the conversion of Ang I to Ang II and Ang-(1-7), respectively. To investigate the role of spinal ACEs in trigeminal neuropathic pain, captopril (an ACE1 inhibitor) or diminazene aceturate (an ACE2 activator) was injected intracisternally on POD 7. The air-puff thresholds were assessed after intracisternal administration of captopril (10 or 100 µg) or diminazene aceturate (1 or 10 μg) and intracisternal injection of Ang-(1-7) (5 or 50 μg) (*n* = 7 animals per group). To identify the participation of the Mas receptor in the antinociceptive effects of Ang-(1-7), A779 (0.1 μg), a selective Mas receptor inhibitor, was injected intracisternally 3 h before the administration of 50 μg of the Ang-(1-7) injection (*n* = 7 animals per group).

#### 4.7.4. Role of the Spinal ACE2 Pathway in Pronociceptive Responses in Naïve Rats

To confirm the role of the ACE2 pathway in pronociceptive processing, DX600 (an ACE2 inhibitor, 5 or 100 ng) was intracisternally injected in naïve rats. After blocking the ACE2 pathway, alterations in the air-puff thresholds were monitored (*n* = 7 animals per group). To investigate the role of the AT1 receptor in the pronociceptive effects caused by blocking the ACE2 pathway, losartan (an AT1 receptor antagonist) was injected 1 h after administering the ACE2 inhibitor. The air-puff thresholds were assessed following losartan injection (*n* = 7 animals per group).

#### 4.7.5. Changes in ACE1 and ACE2 Expression Following IAN Injury

ACE1 and ACE2 expressions in the iTSC were examined on PODs 3, 10, and 50 using Western blot analysis (*n* = 5 animals per group). Moreover, we analyzed the comparison of the ACE1 to ACE2 ratio following IAN injury.

### 4.8. Statistical Analysis

Statistical analyses were performed using SPSS (version 30.0). The normality of the data distribution was assessed using the Shapiro–Wilk test prior to parametric analysis. The behavioral data were analyzed using repeated-measures analysis of variance (ANOVA) followed by Holm–Sidak post hoc tests. Western blot data were analyzed using one-way ANOVA followed by Holm–Sidak post hoc tests. Statistical significance was defined as *p* < 0.05. All data are presented as mean ± SEM.

## 5. Conclusions

**Figure 10:**
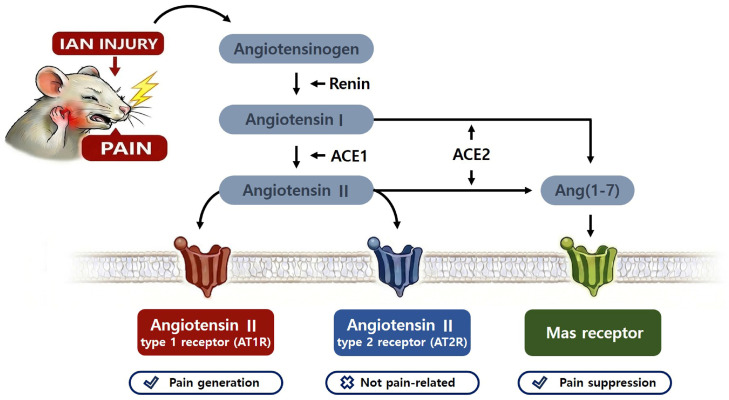
Role of spinal Ang II and its receptors in trigeminal neuropathic pain. Blockade of the spinal ACE1 pathway or activation of the spinal ACE2 pathway attenuated neuropathic mechanical allodynia. The spinal AT1 receptor, but not the AT2 receptor, plays a critical role in pain transmission following nerve injury. These findings suggest that the ACE1 pathway exerts pronociceptive effects, whereas the ACE2 pathway mediates antinociceptive actions in neuropathic pain. Thus, differential modulation of the ACE1/ACE2 axis plays a pivotal role in the development of neuropathic pain following IAN injury.

This study demonstrated that spinal ACE pathways influence neuropathic pain following IAN injury. Blockade of the spinal ACE1 pathway or activation of the spinal ACE2 pathway attenuated neuropathic mechanical allodynia. Pretreatment with a Mas receptor inhibitor blocked the antiallodynic effects induced by Ang (1-7) in rats with neuropathic pain. Pretreatment with losartan blocked the pronociceptive effects induced by blocking spinal ACE2 in naïve rats. Finally, IAN injury increased ACE1 expression and down-regulated ACE2 expression in the iTSC. These findings suggest that the spinal ACE1 pathway has pronociceptive effects, whereas the ACE2 pathway has antinociceptive actions on neuropathic pain. Thus, the differential modulation of ACE1/2 plays a critical role in neuropathic pain after IAN injury, as summarized in Figure 10.

## Data Availability

The raw data supporting the conclusions of this article will be made available by the authors, without undue reservation.

## Abbreviations

The following abbreviations are used in this manuscript:

RAS	Renin–angiotensin system
AGT	Angiotensinogen
Ang	Angiotensin
AT1	Ang II type-1
AT2	Ang II type-2
CGRP	Calcitonin gene-related peptide
ACE	Angiotensin-converting enzyme
Ang-(1-7)	Angiotensin 1-7
CNS	Central nervous system
IAN	Inferior alveolar nerve
iTSC	Ipsilateral trigeminal subnucleus caudalis
ARB	Angiotensin receptor blockers
BBB	Blood–Brain Barrier
